# The Medicare Current Beneficiary Survey (MCBS): Providing Unique Access to Data on the Medicare Population

**DOI:** 10.1093/geroni/igab046.3266

**Published:** 2021-12-17

**Authors:** Hildie Cohen, Sarah Hoyt, Sara Navin, Jennifer Titus, Mia Ibrahim

**Affiliations:** 1 NORC at the University of Chicago, Phoenix, Arizona, United States; 2 NORC at the University of Chicago, Bethesda, Maryland, United States; 3 NORC at the University of Chicago, Chicago, Illinois, United States; 4 NORC at the University of Chicago., Bethesda, Maryland, United States

## Abstract

The Medicare Current Beneficiary Survey (MCBS) is a continuous, multipurpose survey of a nationally representative sample of the Medicare population, conducted by the Centers for Medicare & Medicaid Services (CMS). It collects data on demographics, health insurance, health status, health care expenditures, satisfaction with care, and access to care for Medicare beneficiaries. The MCBS provides a unique source of information regarding beneficiaries aged 65 and over and beneficiaries aged 64 and below with disabilities residing in the United States that cannot be obtained solely through CMS administrative sources. For researchers interested in issues of health care utilization and cost, CMS releases two Limited Data Set (LDS) files for each data year and a Public Use File (PUF) freely available for download and use. Also, special topic based PUFs have been released on the impact of COVID-19 on Medicare beneficiaries. This presentation will demonstrate the importance of the MCBS for research on the Medicare population, discuss how researchers can access the data, where researchers can find published MCBS estimates, what content areas have recently been added, such as food insecurity, limited English proficiency, and COVID-19 vaccination uptake and what new content is on the horizon. The presentation will also discuss the operational challenges posed by the COVID-19 pandemic, and the content enhancement opportunities created by the public health emergency. It will conclude with a review of the suite of materials and documentation available for data users to enhance their research and utilize more timely data.

